# Three-Dimensional Printing Technology in Drug Design and Development: Feasibility, Challenges, and Potential Applications

**DOI:** 10.3390/jpm14111080

**Published:** 2024-10-29

**Authors:** Maria C. Simon, Konstantinos Laios, Ioannis Nikolakakis, Theodore G. Papaioannou

**Affiliations:** Department of Biomedical Engineering and Department of History of Medicine and Medical Ethics, Medical School, National and Kapodistrian University of Athens, 11527 Athens, Greece; smg2200003@uoa.gr (M.C.S.); konstlaios@med.uoa.gr (K.L.); giannisnik@med.uoa.gr (I.N.)

**Keywords:** additive manufacturing, three-dimensional-printed drugs, three-dimensional printing in healthcare, biomedical engineering, personalized medicine

## Abstract

Background/Objectives: The present investigation evaluates the impact of 3D-printing technology on the design of pharmaceutical drugs, considering the feasibility issues and problems concerning technological, pharmaceutical, and clinical matters. This paper aims to review how 3D printing can modify the traditional manufacturing of drugs with personalized medicine-therapy outcomes being individualized and optimized, hence improving patients’ compliance. Methods: The historical development of 3D printing from rapid prototyping to advanced pharmaceutical applications is discussed. A comparison is then made between traditional drug manufacturing approaches and the different techniques of 3D printing, including stereolithography, material extrusion, and binder jetting. Feasibility is assessed based on clinical trials and studies evaluating the efficacy, safety, bioavailability, and cost-effectiveness of 3D-printed drugs. Results: Current evidence indicates that material selection, regulatory barriers, and scalability issues are some of the major challenges to be overcome for wider acceptance. Other matters, such as ethical issues concerning patient data privacy, the misuse of 3D-printing technology, and technical complexities related to pharmaceutical 3D printing, are discussed further. Future applications also include bioprinting and in situ printing together with their implications for personalized drug delivery, which will also be discussed. Conclusions: This review stresses that intersectoral collaboration and the updating of regulatory frameworks are a must to overcome the barriers that confront 3D-printing applications in drug development. can could be an opportunity for innovative licensing and manufacturing techniques in pharmaceutical product development that can change the paradigm of personalized medicine through modern printing techniques.

## 1. Introduction

Is it imaginable that, in the future, everyday pharmaceutical drugs could be produced with a 3D printer on demand, eliminating the need for pharmacies? This question explores the feasibility, challenges, and potential applications of 3D printing in pharmaceutical drug design.

Our era is characterized by fast-rising technical developments and groundbreaking innovations. Among these advancements, 3D-printing technologies and pharmaceuticals have emerged as domains of considerable promise and potential. Three-dimensional printing has a vast, unrealized potential for use regarding personalized medicine by the formulation of drugs to the needs of each patient. Personalized medicine requires tailoring treatment according to the individual characteristics of the patient, while 3D printing can eventually provide drugs differentiated by dose, shape, and release profile. One example demonstrated the feasibility of 3D printing for the fabrication of tablets, including those with differentiated dosages according to the specific needs of the patient, such as polypills containing multiple drugs for elderly patients to increase compliance and reduce medication burden [1]. Further, 3D printing has been used for the fabrication of fast-dissolving orodispersible films, particularly for patients who have difficulty swallowing [2]. These applications highlight some of the unique capabilities of 3D printing in addressing individual patient needs. A pharmacist across various healthcare settings is always at the forefront of designing and dispensing 3D-printed medication to patients, ensuring effective care through rigorous customization [3]. Can 3D printing truly revolutionize drug design?

Three-dimensional printing, known as additive manufacturing, represents a technological breakthrough that enables the creation of three-dimensional objects from digital models by layering materials [4]. In the pharmaceutical industry, 3D printing signifies a paradigm shift, offering opportunities for streamlined prototyping, the formulation of complex drug structures, and a pathway toward personalized medicine [4,5].

**Figure 1:**
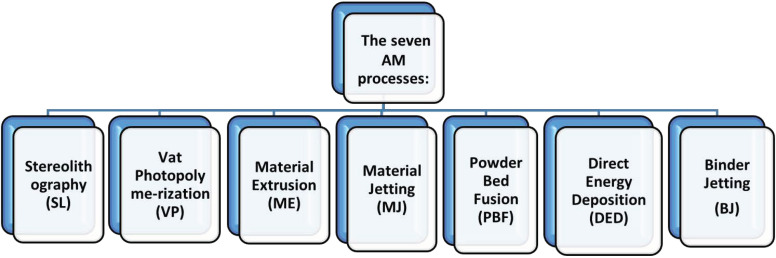
The seven fundamental AM 3D-printing applications.

Monteiro et al. outline seven types of 3D-printing applications, dependent on the materials and printing requirements, each with its advantages and limitations [6]. Moreover, novel 3D-printing materials, such as smart materials, ceramics, electronics, biomaterials, and composites, are shaping the prospect of pharmaceutical manufacturing [7]. Among these applications, our focus in this paper is on some key elements of new derivates based on seven fundamental AM (additive manufacturing) 3D-printing technologies (Figure 1). These encompass stereolithography (SL), which employs ultraviolet light to cure the liquid resin layer for the formation of a solid object. Vat photopolymerization (VP) is responsible for solidifying liquid resin using ultraviolet light emitted from a vat, producing multiple layers. Material extrusion (ME) is a process that involves pushing bond-ready material through a heated nozzle in order to deposit it in successive layers, while material jetting (MJ) selectively deposits droplets of material onto a substrate. Powder bed fusion (PBF) selectively fuses layers using a laser or electron beam, whereas direct energy deposition (DED) melts the material and deposits it layer by layer using a focused energy source. Finally, binder jetting (BJ) deposits a binding agent onto a bed of powder to create layers [7].

As we navigate these innovations, we aim to elucidate the foundational principles underlying the use of 3D printing in pharmaceuticals, delineating the various processes, materials, and types of 3D printing that are applicable to drug design. This investigation examines the history of 3D printing, traditional methods of drug manufacturing, and the fundamental concepts. Additionally, we evaluate the feasibility of 3D-printed drugs by considering the existing studies and uncovering the challenges spanning technical, regulatory, and ethical dimensions that hinder the widespread adoption of 3D printing for pharmaceuticals.

Exploring the potential applications of 3D printing in personalized medicine, drug matrix formulation, and dosage customization, we aim to highlight the efficiency of 3D-printed drugs through exemplary case studies. A comparative analysis of 3D-printing and traditional drug manufacturing methods will elucidate this innovative approach’s transformative potential and inherent advantages, including the current guidelines and challenges of obtaining regulatory approval.

Finally, we examine the future directions of 3D printing in drug design, emphasizing the evolution of drug development and further possibilities, like organ printing.

## 2. Methodology

We performed a non-systematic literature review aiming to provide the most up-to-date information regarding three-dimensional printing technology in drug design and development, highlighting the feasibility, challenges, and potential applications of this technology. The review also focuses on the potential impact of the 3D printing of drugs on the pharmaceutical industry and personalized medicine. For the literature search, we used the databases Medline/PubMed, Google Scholar, and Science Direct using the search terms “3D printing”, “three-dimensional printing”, “3D bioprinting”, “3D printed drugs”, “additive manufacturing”, and “drug delivery systems”. We focused on journal articles published in the English language between the years 2000 and 2023. Theoretical treatments and experimental studies were covered, in general, with an interest in both from a drug development perspective.

## 3. The History of 3D Printing

It is important to mention that, during the invention of 3D printing in the industry, individuals pursued patents and pioneered innovations that led to the diverse applications of 3D-printing techniques known today. However, rapid prototyping was one of the earliest additive manufacturing techniques, enabling the rapid creation of prototypes to accelerate the testing of product viability before market introduction.

It is worth mentioning that, in 1859, a French “photosculptor” named François Willème demonstrated the world’s first “3D scanning” technology by using 24 cameras to simultaneously photograph a subject from different angles. A few years later, in 1892, inventor Joseph E. Blanther was awarded a patent for a method of creating 3D topographical maps using a layering method, similar in concept to today’s 3D printers.

Milestones in 3D-printing development from the early 1960s to the early 2000s in both the industry and medical systems [8] include:

o The University of Battelle Memorial Institute in Ohio explored using photopolymers to create 3D-printed objects during the 1960s.
o The invention of solid photography by the Dynell Electronics Corporation. This technology aimed to cut cross-sections based on a computer model, which represents one of the main 3D-printing-stage principles during the 1970s.
o Hideo Kodoma from the Nagoya Municipal Industrial Research Institution in Japan published the principles for the automation of 3D models using photosensitive resin and rays. These were the first approaches toward stereolithography in 1980–1981.
o Stereolithography (SLA) was invented in 1984. The first stereolithography patents by Alain Le Méhauté, Olivier de Witte, Jean Claude André in France, and Charles ‘Chuck’ Hull existed in the USA
o Dr. Hideo Kodoma patented the SLA invention in 1986.
o The first commercial SLA printer in the world was produced by 3D Systems in 1988.
o Scott and Lisa Camp founded “Stratasys” in 1989. They filed a patent for the formation of rapid prototyping, laying the groundwork for the first principles of fused deposition modeling (FDM).
o Hans Langer formed the company electro-optical system (EOS) in late 1989, which made the fabrication of 3D parts directly from computer design models possible.
o Carl Deckard developed the concept of the selective laser sintering process (SLS). The process consisted of a selective solidification of powder using a laser beam to fuse powdered materials layer by layer.
o The 3D-printing industry split into two branches in the early 1990s: one focused on engineering complex parts, and the other on concept development and functional prototyping.
o By the late 1990s, three companies remained in the 3D industry: Stratasys, 3D systems, and EOS.
o The first production of SLS printers occurred in 1992.
o Deckard founded “Sinterstation” in 2000 launching SLS technologies into the industry.
o In the early 2000s, 3D printing gained interest and importance in the medical field. Oral fast-disintegration tablets, such as the FDA-cleared Spritam^®^ (Levetiracetam), have been fabricated by SLA at Aprecia Pharmaceuticals, Blue Ash, Ohio, USA, while other ME techniques have been used for the fabrication of scaffolds and implants loaded with drugs intended for controlled release [9,10,11].
o After 2010, advancements in bioprinting and drug-loaded implants occur. Cinnarizine is formulated by 3D-printing technology into a gastroretentive dosage form [12].
o In 2003, Dr. Thomas Boland filed the first patent for a technique that involved the printing of viable cells.
o In 2004, Gabor Forgacs patented a scaffold-free bioprinting technique that enabled the simultaneous printing of multiple cells.
o In 2005, 3D-printed hydroxyapatite scaffold designs, based on anatomical information from individualized patient images, emerged.
o With the advancement of FDM technology by Stratasys in 2005, two open 3D-printer projects came to life: Fab@Home and the RepRap Movement. The goal of both projects was to make 3D-printer designs affordable to a wider audience.
o In 2007, the first RepRap 3D printer, named “Darwin”, was released, followed by the later versions of “Mendel”, “Prusa Mendel”, and “Huxley”.
o In 2009, Organovo was awarded the first NIH grant for bioprinting vessels.
o Engineers at the University of Southampton in the U.K. designed the world’s first unmanned 3D-printed aircraft. The total cost was less than USD 7000.
o In 2015, the Swedish company Cellink released for sale the first commercial bio-ink. It is made from nanocellulose alginate, a material derived from seaweed, and can be used for printing tissue cartilage.
o FDA approval of Spritam (the first 3D-printed drug) in 2018.
o In 2018, a gastric floating system was developed with riboflavin, showing an excellent floating ability of up to 3 days. This was a new way of achieving optimum drug release and thus showed this by merging different 3D-printing techniques; new frontiers were established for generating sophisticated drug delivery systems (Fu et al., 2018) [13].
o Emerging developments in bioprinting also led to the fabrication of tissue scaffolds able to deliver drugs right to the disease sites, with greater potential for effectiveness in applications within regenerative medicine [14,15,16].
o Advances in drug-loaded implants and bioprinting in 2023.

**Figure 2:**
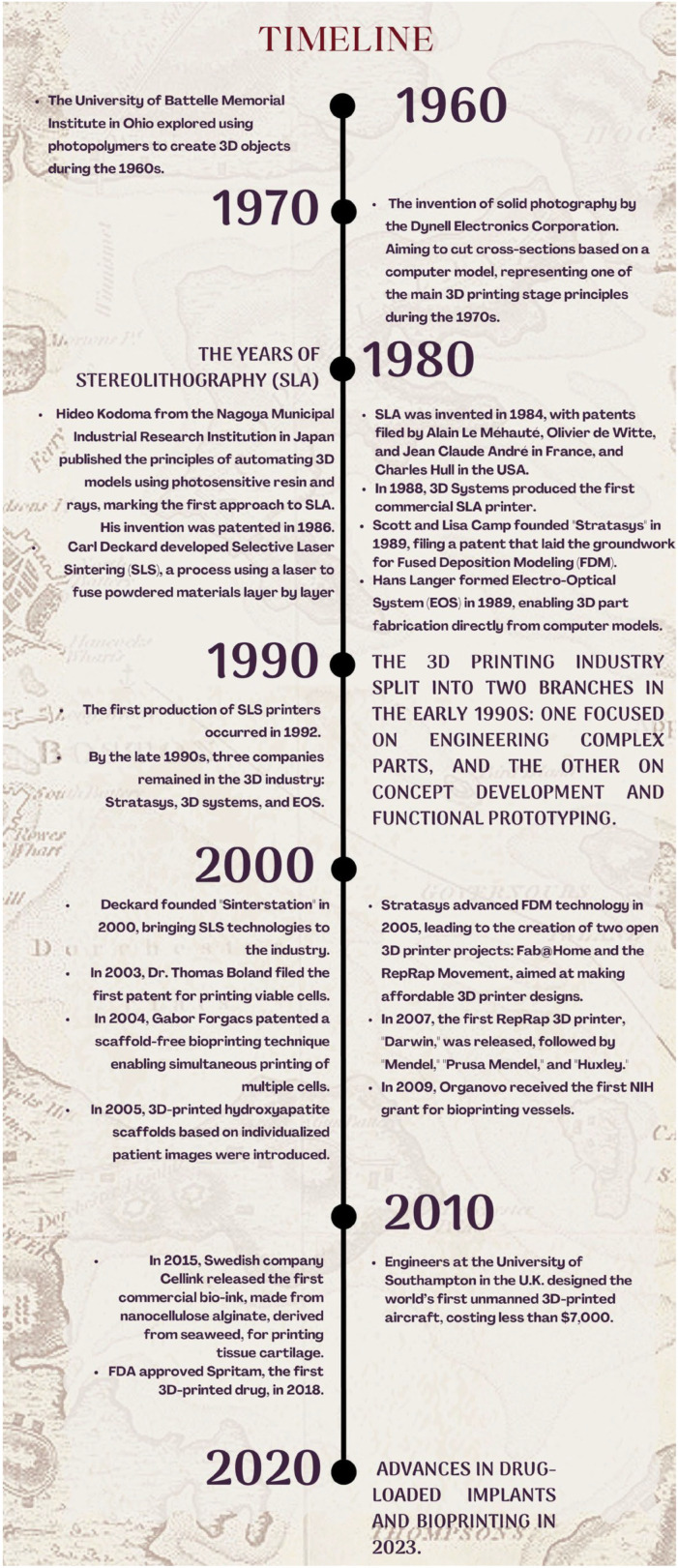
Brief historical overview of 3D printing.

To provide a concise overview of the key milestones in the development of 3D printing technology, a timeline summarizing the historical progression from the 1960s to the present has been included in Figure 2. 

### Charles W. Hull—The Pioneer of the 3D-Printer Industry [17]

Charles W. Hull is a well-known inventor and engineer recognized for his innovations in the creation of 3D-printing technology. Hull is considered one of the pioneers of 3D printing for his major contribution to the field through the invention of stereolithography (SLA). Hull’s work initially involved using UV light to harden tabletop coatings, which inspired his use of UV light in the SLA process. The first object he built using this technology was a small cup, 5 cm tall. In 1984, he co-founded 3D Systems, which gained popularity in the automobile and aerospace industries and was widely used in medical applications [8].

## 4. Basic Principles and Methods—The Traditional Methods of Drug Manufacturing

The future of medicine appears to be shifting toward personalized dosage regimens, tailored to individual metabolic profiles [1]. Advances in gene sequencing offer promising avenues for enhancing drug delivery efficiency [16], highlighting the significant variability among individuals.

Among various technological innovations, additive manufacturing (AM), or 3D printing, is especially emerging in pharmaceutical applications, aiming to offer personalized solutions. This technique involves translating computer-aided design (CAD) models into physical objects through layer-by-layer construction. Its versatility has elevated its significance in drug delivery over the past decade [18].

Traditional drug delivery methods include oral drugs, scaffolds, transdermal patches, rectal or vaginal delivery, implants, and intravenous devices [18].

Material extrusion, such as fused deposition modeling (FDM), stands out as one of the most cost-effective methods for printing drug delivery systems. It processes a wide range of materials, including thermoplastics, waxes, gels, pastes, and clay, allowing for versatile fabrication. FDM operates by heating an input filament to a molten state and depositing it layer by layer onto a printing platform using a moving nozzle. This method is applied for oral tablets, implants, scaffolds, iodine delivery, rectal/vaginal delivery, transdermal patches, meshes, and catheters [18].

Vat polymerization techniques, such as stereolithography (SLA) and digital light processing (DLP), offer high-resolution printing capabilities. SLA utilizes a UV-light source to solidify liquid resin layer by layer, while DLP uses visible light to achieve the same result. Although these techniques have fewer compatible polymers, they excel in producing drug delivery channels loaded with various drugs. SLA and DLP can be used for fabricating oral drugs, scaffolds, and transdermal patches [18].

Binder jetting/inkjet printing (IJ) deposits ink on a powdered bed, creating layers that bind together to fabricate oral tablets, orodispersible films (ODFs), and implants. Notably, this technique was used to produce the first FDA-approved 3D-printed drug with ZipDose^®^ technology. ZipDose^®^ technology is a platform that allows for the rapid disintegration of high-dose medications in a small volume of water. Its applications include oral tablets, orodispersible films (ODFs), and implants [18].

Table 1 provides a comprehensive comparison of the various 3D-printing techniques employed for the development of pharmaceuticals. Their applications include oral drugs, implants, scaffolds, and transdermal patches, and the special features of each process.

Direct energy deposition (DED) techniques, while limited by material availability and accuracy, still have potential for printing drug-loaded implants and scaffolds. DED involves locally melting a powdered or filament material using a laser or electron beam and then depositing it layer by layer onto a heated substrate [18].

Powder bed fusion encompasses techniques, like selective laser sintering (SLS) and selective laser melting (SLM), which fuse powdered materials layer by layer to create solid objects. These techniques are valued for their accuracy and strength, making them suitable for implants and scaffolds in drug delivery systems.

Material jetting (IJ) involves jetting droplets of material onto a substrate and solidifying them, typically using UV light. It offers a high-resolution and surface finish, making it suitable for fabricating oral tablets and transdermal patches.

Sheet lamination builds objects by stacking layers cut from sheets of material. While less commonly used for pharmaceutical manufacturing, it can be suitable for certain drug delivery applications where layer-by-layer construction is advantageous.

Ensuring regulatory compliance presents substantial challenges in the additive manufacturing of pharmaceuticals. In this respect, clear regulatory pathways are needed for the full integration of 3D printing into clinical settings to guarantee the quality and safety of 3D-printed medications. In this context, it is essential to address specific challenges in material selection, printability, and scalability, which are crucial for the wider adoption of these technologies [3]. Issues, such as drug degradation, improper loading percentages, and toxic reactions, necessitate rigorous in vivo testing for biocompatibility and suitability. Regulatory approval from bodies like the FDA is mandatory before commercialization. Draft guidelines issued by the FDA in 2016 provide a regulatory framework, emphasizing the importance of in vivo, in vitro, and clinical evaluations for printed drug forms [18].

The National AM Innovations Cluster (NAMIC) in Singapore and similar initiatives worldwide highlight the growing emphasis on regulating additive manufacturing in the pharmaceutical sector. Forecasts indicate significant revenue growth in additive manufacturing, underscoring the importance of addressing regulatory and legal challenges to ensure safe and effective drug delivery systems [18,31,32].

## 5. Applications of 3D Printing for Pharmaceuticals

The strategic integration of 3D-printing technology in pharmaceutical manufacturing promises extensive advancements, aligning medication formulations precisely with individual patient needs. The importance of 3D printing lies in its ability to revolutionize drug dosage forms and enhance therapeutic outcomes through tailored drug delivery mechanisms.

The integration of 3D printing in pharmaceuticals presents revolutionary steps in tailoring drug formulations to patients’ needs, which can improve therapeutic outcomes through personalized drug delivery mechanisms. Various 3D-printing technologies, including powder-based, extrusion-based, inkjet-based, and laser-based methods, enable the precise creation of complex drug delivery systems and formulations. For example, 3D printing facilitates the development of child-friendly formulations, such as chewable tablets with specific dosages and flavors. It also improves compliance among pediatric patients. Additionally, integrating 3D printing with artificial intelligence can enhance quality control and customization, ushering in a new era of digital pharmacy [3].

For instance, powder-based methods, such as selective laser sintering, fuse powdered materials layer by layer to provide robust and accurate drug delivery devices. Inkjet printing of indomethacin-loaded transdermal films resulted in promising drug release and permeation properties, making it a suitable technique for personalized transdermal medication [33]. Extrusion-based methods, like fused deposition modeling, are associated with the use of heated filaments to build an object layer by layer and provide cost-effective and versatile solutions for drug fabrication. Furthermore, inkjet printing was successfully used for the preparation of amitriptyline hydrochloride tablets, showing promising drug release profiles combined with effective drug loading [34]. Selective laser sintering was also applied to manufacture paracetamol tablets that exhibited strong structures and pH-independent drug release, with no evidence of drug degradation during this process [35]. Further development using SLS technology resulted in orally disintegrating paracetamol tablets with enhanced drug release profiles, optimized through laser scanning speed for a rapid onset of action in patient care [36]. Additionally, stereolithography technology was used for the printing of drug-loaded hydrogels, including ibuprofen-loaded hydrogels. This resulted in higher drug release due to the high water content in the hydrogel, demonstrating its promising potential in personalized drug delivery systems (Martínez et al., 2017) [37]. In another study, SLA technology was used to fabricate hydrogels loaded with ascorbic acid. Hydrogel structures with geometric shapes demonstrated controlled release characteristics, with the highest release rates observed in honeycomb and coaxial annulus geometries, further demonstrating the use of SLA technology for drug delivery applications (Karakurt et al., 2020) [38]. Inkjet-based methods, such as binder jetting, involve depositing droplets of liquid onto substrates to create complex drug structures, as seen in the FDA-approved 3D-printed drug, Spritam (Levetiracetam). Laser-induced methods, which include stereolithography, use UV light for the solidification of liquid resins and allow for high-resolution printing of oral drugs and implants.

Different PBF techniques have been developed into various drug delivery devices of high accuracy, including, but not limited to, implants, for localized release. Inkjet printing of ketoprofen-loaded buccal films demonstrated excellent drug release and permeation, proving its potential in personalized drug delivery systems [39]. Additionally, orodispersible tablets offer advantages, such as fast drug disintegration and easy swallowing, which help pediatric and geriatric conditions. Research on ondansetron orodispersible printlets showed rapid disintegration in less than 15 s, with more than a 90% released within five minutes, demonstrating its quick therapeutic response [40]. Implants created using the PBF technique can be designed to deliver drugs at the disease site, offering localized treatment while limiting systemic exposure. Three-dimensional printing of paracetamol tablets achieved a zero-order release profile, which may provide more consistent in vivo drug release and enhance the therapeutic outcomes for chronic diseases [41]. This is particularly valuable for cancer treatment, where precision and minimal side effects are critical. Additionally, PBF enables the creation of complex geometries with internal channels that can house multiple drugs, allowing for controlled release over time, which is particularly needed for diseases requiring chronic treatment [21].

Several investigations have focused on bioprinting medication-loaded, patient-specific scaffolds for tissue regeneration. three-dimensional printing methods have also applied inkjet printing to several biologics. Inkjet printing of lysozyme onto buccal films was successfully performed without compromising mechanical or mucoadhesive properties, showing the effectiveness of inkjet printing in making buccal films for the delivery of biologics [17]. Moreover, the inkjet printing technique has been applied to prepare the oromucosal dosage form. Lidocaine hydrochloride and piroxicam were printed successfully onto fibrous matrices and showed good drug entrapment and solidification. The printed drug closely matched the theoretical dose, demonstrating the accuracy of 3D-printing technologies in drug delivery systems [42]. Scaffolds can be designed to release numerous bioactive agents, such as growth factors and antibiotics, thereby enhancing tissue regeneration or preventing infection. Tissue engineering makes use of scaffolds to support cellular ingrowth, while bioprinting allows the deposition of materials with defined precision and, as such, builds matrices that are similar to the native ECM. A case in point is the use of scaffolds in bone regeneration, where 3D-printed scaffolds loaded with BMPs have shown considerable improvement in healing times and bone density. Moreover, bioprinting is being studied for its potential application in producing scaffolds with controlled drug release to support long-term tissue recovery [43].

Despite these innovations, the field faces considerable technical and regulatory challenges. Regulatory frameworks need to catch up to accommodate the specific characteristics of 3D-printed pharmaceuticals and ensure the safety and efficacy of these products through new guidelines. Scaling up introduces its own set of challenges, requiring development to bridge the gap between prototype innovation and mass production [14,18,22].

Fundamental to this innovation is a spectrum of materials selected for their biocompatibility, solubility, and mechanical properties. Materials, such as polylactic acid (PLA), polyvinyl alcohol (PVA), and hydroxypropyl methylcellulose (HPMC), emerge as frontrunners, facilitating the formulation of implants, scaffolds, and drug delivery systems with unparalleled precision and safety [2,25,26].

The advantages of 3D printing expand beyond material selection. Its fundamental ability to customize dosage forms, intricate geometries, and drug-release profiles marks a new era of patient-centric healthcare. For instance, the fabrication of polypills tailored to individual medication regimens exemplifies 3D printing’s potential to streamline treatment protocols and enhance patient adherence [10]. Specific examples include the design of a multi-layered polypill containing six drugs using a novel stereolithographic method, which allows for better control of the drug release from the polymeric matrices and improves patient compliance through the consolidation of several medications into one dosage form [30]. Another example is the five-in-one combination polypill with defined immediate and sustained release profiles, demonstrating that 3D printing can be used to create complex tablets containing multiple drugs to treat different conditions with varying kinetic requirements simultaneously [9].

Moreover, 3D printing catalyzes breakthroughs in the development of medical devices, particularly focusing on implantable drug delivery systems. One significant example is the development of 3D-printed biodegradable stents for cardiovascular applications. Such stents can locally deliver drugs to the site of implantation with controlled release and reduced systemic side effects, as seen with antibiotics for treating osteomyelitis, where structural support is combined with localized delivery.

It has also enabled microneedle production in pain-free drug delivery. One notable example is the delivery of cisplatin using 3D-printed microneedles. In this study, microneedle arrays were fabricated, demonstrating good release rates and effective anti-cancer activity, proving the potential of microneedles in targeted chemotherapy delivery [44]. These microneedles are designed to dissolve and release the drug directly into the bloodstream after penetrating the skin. By combining drug therapy with patient-specific implants created through 3D printing, this approach offers optimized treatment efficacy, comfort, and safety for patients [18,19,20].

However, the promise of 3D printing is met by regulatory and technical challenges. The regulatory framework is always behind technological development, and it needs comprehensive guidelines to set up the quality and safety measures for 3D-printed pharmaceuticals. For example, the FDA has published guidance for 3D-printed medical devices, but it has not yet fully addressed pharmaceuticals leaving a gap in regulatory oversight that is clouding the approval process for new 3D-printed drugs. Additionally, guidelines are currently being formulated by the European Medicines Agency and other international regulatory bodies, but comprehensive standards have not yet been implemented. Moreover, 3D-printing technologies evolve so rapidly that existing regulations quickly become outdated, requiring continuous updates to regulatory frameworks to keep pace with technological advancements. Additionally, large-scale production presents another obstacle; several steps must be taken to bridge the gap between innovation and mass production [1,6,18].

## 6. Feasibility—The Effectiveness of Printed Drugs

We now explore the effectiveness of printed drugs, looking at their efficacy, safety, bioavailability, long-term outcomes, and cost-effectiveness. It enhances efficiency and cost-effectiveness in pharmaceutical manufacturing. By allowing on-demand production and reducing wastage, it reduces time and costs incurred in production, hence forming a sustainable alternative to traditional techniques [45]. Based on various clinical trials and studies, we gain insights into the therapeutic outcomes and impact of 3D-printing technology on pharmaceutical development.

Starting with efficacy and therapeutic outcomes, one example is the FDA’s approval of Spritam (Levetiracetam) in 2018 [11]. Ongoing research emphasizes the potential for tailored medication formulations, especially for pediatric and geriatric patients, ensuring optimized therapeutic impact through dimension-specific product designs [11].

Clinical trials of 3D-printed pharmaceuticals have presented promising results. A pediatric patient trial showed the efficacy of orodispersible film, which was printed using binder jetting technology, in improving adherence [2]. Another trial that tested drug-loaded implants for localized cancer treatment showed improved targeting of drugs with reduced side effects [14]. A clinical trial in gastroretentive floating tablets further demonstrated the capability for zero-order drug release and, hence, more precise and regular chronic medication. Three-dimensional printing was used to manufacture floating tablets in this trial, presenting a significant increase in bioavailability with improved compliance in patients [28]. Another approach is the polypill, whose stereolithography-manufactured, multi-layer, 3D-printed system contained six drugs, evidencing its potential in the consolidation of complex medication regimens. This has improved patient compliance, especially among the elderly, as fewer doses are taken per day [30]. Three-dimensional printing influences drug release kinetics, since some studies revealed that binder volume affects drying time and residual solvent release, while powder and ink properties and porosity variations influence product quality attributes. Also, observations of drug dissolution profiles designate the impact of layer height and scale count on release kinetics [11].

It is crucial to acknowledge the advantages and limitations of 3D-printing technology in terms of the assessment of the safety profile and tolerability of printed drugs. Regulatory oversight is essential to ensure the safety and effectiveness of medication production, given the variables affecting the efficiency and health of computationally engineered dosage forms [11].

Regarding bioavailability and pharmacokinetics, the SLA method allows for flexibility in object geometry and porosity, resulting in fast-disintegrating dosage forms without the need for binding agents. The approach of SLS, on the other hand, offers advantages for strong dosage formulations [11].

Concerning the long-term outcomes and approval status, Spritam stands as a pioneer in commercially available pharmaceutical drugs authorized by the US FDA [9] and represents a significant milestone in the integration of 3D printing into pharmacotherapy. While legislative decisions are increasingly focused on advancing science and technology, 3D printing remains a key approach in pharmaceutical development [11].

In terms of cost-effectiveness and healthcare economics, 3D printing demonstrates sustainable advantages over conventional manufacturing methods, revolutionizing drug production and distribution. With the global 3D-printing market projected to have a substantial economic impact by 2025, pharmaceutical companies can adopt this technology to enhance efficiency and accessibility [11]. The effectiveness of printed drugs encompasses a complex evaluation spanning efficacy, safety, bioavailability, long-term outcomes, and economic considerations.

## 7. Challenges of 3D Printing Drugs

Despite the promising opportunities presented by this technology, various technical and regulatory difficulties obstruct its widespread implementation in the pharmaceutical and healthcare sectors [46].

One significant challenge lies in the selection of suitable materials for the 3D printing of drugs. Factors, such as biocompatibility, stability, and regulatory approval, must be carefully considered to ensure the safety and efficacy of the printed formulations. In addition, issues related to printability, including nozzle clogging, layer adhesion, and print accuracy, pose significant obstacles that can affect the quality and consistency of printed drugs.

Another remarkable challenge is to achieve uniform drug dosage within printed formulations. Enhancing dose consistency and accuracy is essential to meet regulatory requirements. Scalability, particularly in the mass production of drugs, remains a concern. Addressing issues such as production time, cost-effectiveness, and regulatory compliance on a larger scale is essential for the widespread adoption of 3D printing in pharmaceutical manufacturing.

Regulatory barriers further hinder the approval of 3D-printed drugs. The lack of specific regulatory guidance for drug production using 3D-printing technology poses a problem to its implementation in the healthcare system. While the FDA has issued regulations for the use of 3D printing in medical devices and prosthetics, drug production remains unexamined [46].

Intellectual property issues also present challenges in the 3D printing of drugs, including patent infringement, technology licensing, and protection of proprietary formulations. These legal considerations add complexity to the development and commercialization of 3D-printed pharmaceuticals.

Technological factors, such as the use of heat, solvents, and light in 3D printing processes, may affect the stability and quality of printed drugs [45]. In post-printing products, essential steps to overcome are the challenges of quality control and developing reliable evaluation methods. Non-destructive techniques, such as NIR and Raman spectroscopy, offer promising solutions for the real-time evaluation of drug product quality at production sites, such as clinics and hospital pharmacies [46].

While various types of 3D printers have been explored for pharmaceutical dosage form production, ensuring compliance with good manufacturing practice (GMP) standards remains a challenge. Efforts are being made to develop compact printers that meet GMP requirements, with companies like FabRx taking the initiative [46].

## 8. Issues/Limitations of 3D Printing and Future Potential Applications

Currently, 3D printing encounters several limitations that shape its present applications. Particularly, while the technology enables the creation of pill molds and direct printing using drug powders as raw materials, it battles with challenges inherent in different printing technologies, like FDM, SLA, and SLS [14].

Despite its current limitations, the future holds promise for 3D printing, particularly in pharmaceuticals. Three-dimensional printing in personalized medicine envisions the customization of nutritional products, organs, and drugs. It is expected that this tendency will spread throughout pharmacy settings, potentially altering the production and delivery of pharmaceuticals. With the advent of on-demand drug printing, pharmacies can receive medication formulations via email, leading to cost-effectiveness and increased patient-centric healthcare solutions [47].

Another noteworthy advancement to mention in 3D-printing technology is the bioprinting of complex organs. Progress in printing vascular networks bodes well for the potential fabrication of viable organs. Some breakthroughs include the successful fabrication of complex tissues, such as liver and kidney tissues, opening the door for live implants and tissue models for drug discovery. In the future, stem cells taken from deciduous teeth can be used as a source of stem cells to develop new tissues and organs [47].

Innovative trends, like in situ printing and implants printed within the human body during surgeries, hold promise for precise lesion repair. Utilizing 3D bioprinting enables the deposition of cells, growth factors, and biomaterial scaffolds to repair internal and external organs. Advances in portable 3D printers and robotic bioprinters for in situ tissue repair signal a combination of precision and efficiency to redefine the future of medical intervention [47].

## 9. Ethical Considerations

Integrating 3D-printing technology into pharmaceutical drug development raises many questions and pitfalls that need to be addressed.

Ethical challenges in personalized medicine are significant. The primary concerns involve the secure storage and management of patient data used to personalize drugs [7]. Lee (2005) highlights the ethical and social challenges surrounding pharmacogenomics and personalized medicine, emphasizing the critical importance of securing patient data due to their sensitivity and implications for patient privacy and consent [16]. Other issues include the equitable distribution of 3D-printing technologies, ensuring they do not exacerbate existing healthcare disparities among underserved populations [32].

These matters primarily concern the ethical implications of patient data privacy and public access to 3D-drug-printing methods, which could lead to misuse. The customization of drugs through 3D printing relies heavily on detailed patient information, including genetic data, medical history, and specific health needs. Personal health data are essential for creating personalized medications that meet individual requirements. The collection, storage, and use of such information raises concerns about privacy and data security. Protecting patient data from unauthorized access or breaches is crucial. In the event of a personal data breach, skepticism and negativity toward the medical community could give rise to new conspiracy theories, potentially undermining public trust in healthcare institutions. One of the main questions that also arises is the ownership of the data. Guidelines on patient data ownership are essential to ensure that patients retain control over their personal health information, used solely for their benefit unless agreed otherwise. For the medical community, a prime target would be ensuring that the benefits of 3D printing are distributed at an affordable cost, to make the technology accessible to a great part of the world’s population. The industry should aim to make 3D-printed drugs affordable. The potential for misuse of 3D-printing technology in non-pharmaceutical industries is also concerning. The ability to print narcotics on demand opens the door to the unauthorized production of dangerous substances. This could lead to serious public health risks, as individuals might access harmful drugs without proper regulation, potentially creating a black market for 3D-printed narcotics. Establishing regulatory frameworks to prevent the misuse of 3D-printing technology without stifling innovation is vital.

## 10. Discussion

Three-dimensional printing can introduce breakthrough developments in pharmaceutical drug design. Key advantages of by 3D printing, for which many benefits can be derived by patients, are personalizing the formulation of drugs and customizing dosages. Each of these opportunities is tempered by various technical, regulatory, and ethical challenges if 3D printing is to achieve mainstream use. The effective integration of 3D printing in pharmaceutical manufacturing, on the other hand, depends on technological advancement, clear-cut regulatory frameworks, and ethical concern resolutions.

The FDA’s approval of Spritam (Levetiracetam) in 2018 created a new opportunity for personalized medication, particularly for pediatric and geriatric patients, through the use of 3D-printing techniques. Another important aspect is that 3D printing can affect the kinetic properties of drug release. Key factors of 3D printing, such as binder volume, powder properties, and porosity, determine the quality and functionality of drugs.

These AM 3D-printing technologies have revolutionized drug design by making personalized formulation possible targeting patient’s specific needs. Manufacturing techniques, such as SLA and FDM, allow the creation of elaborate dosage forms, like polypills and extended-release profiles. Such innovations are imperative in the management of chronic conditions where the precision of dosage and timing is crucial for optimized therapeutic outcomes. Bioprinting technologies offer drug creation upon demand in rare diseases as well [10,22].

Even with these developments, technical challenges are still present. Material selection, printability issues, and quality control are major problems. Homogeneity of drug dosage in the printed formulations is very important for regulatory compliance and a desired therapeutic outcome. Moreover, issues concerning scalability, mainly related to mass production, should be tackled if 3D printing is going to be applied in pharmaceutical manufacturing.

Currently, considering regulatory barriers, very few 3D-printed pharmaceuticals have been approved. While the FDA has issued guidelines on 3D-printed medical devices, there is a lack of good regulatory surveillance for pharmaceuticals, which engenders problems in the approval process for new 3D-printed pharmaceutical drugs. While the EMA, and other international regulatory bodies, have guidelines in various stages of development, no comprehensive standards have been adopted to date.

The impact of 3D printing on healthcare systems and patient outcomes is very promising. It has the potential to revolutionize drug production, distribution, and patient care, from facilitating personalized medicine and optimizing drug delivery mechanisms to increasing the effectiveness of treatments. However, a collaborative platform among the stakeholders to achieve full potential of 3D printing in healthcare is necessary.

Emphasis on establishing regulatory frameworks should be made to make sure that the 3D-printed pharmaceuticals are safe and effective. Encouraging innovation through investment in research is another essential factor. In particular, the ethical concerns related to the use of personal patient data and issues of consent will need consideration regarding the adoption of 3D-printing technology in healthcare.

## 11. Future Directions

We anticipate further progress of 3D-printing technology, parallel to the changes in regulations and healthcare requirements. Bioprinting and in situ printing are emerging technologies that can expand the use of 3D printing in pharmaceuticals. Consistent regulatory updates, such as refining guidelines for 3D-printed pharmaceuticals, will be vital in shaping the field’s direction. We still need to overcome scalability issues and promote interdisciplinary collaboration to maximize the benefits of 3D printing in healthcare.

## 12. Conclusions

Current technologies in additive manufacturing (3D printing) drive the revolution in drug design and development. For the first time, there are new opportunities to consider each patient’s needs individually, which is the case for chronic and complex diseases requiring specific treatments. As the regulation frameworks continue to improve and overcome technical barriers, 3D printing in design and manufacturing will continue to play an increasingly important role in personalized medication.

## 13. Patents

There are no patents resulting from the work reported in this manuscript.

## Figures and Tables

**Table 1:** Three-dimensional-printing applications for drug development.

3D Printing Technology	Applications Examples	Characteristics	References
Stereolithography (SLA)	Oral drugs (discovery phase) Implants (clinical phase)	High-resolution printing uses UV light to cure liquid resin layer by layer Ideal for complex structures	[9,18,19]
Vat Photopolymerization (VP)	Used in scaffolds and transdermal patches during the discovery phase, moving into the clinical phase for implants	Uses UV light emitted from a vat to solidify liquid resin High precision Limited to photopolymers	[18,20]
Material Extrusion (ME)	Oral tablets Implants Scaffolds Transdermal patches (discovery phase)	Cost-effective Versatile Uses heated filaments Suitable for various materials (thermoplastics, waves, and gels)	[18,20,21]
Fused Deposition Modeling (FDM)	Oral tablets Scaffolds Implants	Uses heated thermoplastic filaments Cost-effective Widely accessible Suitable for rapid prototyping	[18,21]
Digital Light Processing (DLP)	Oral drugs Scaffolds Transdermal patches	Uses visible light to cure liquid resin High resolution Faster than SLA and is ideal for fine details	[18,20]
Binder Jetting (BJ)	Oral tablets (commercial phase) Orodispersible films Implants	The first FDA-approved 3D-printed drug (Spritam) Uses a liquid binder to fuse powder layers Customizable dosages	[2,10,18,20]
Direct Energy Deposition (DED)	Implants (still in the discovery phase) Scaffolds	Melts and deposits material layer by layer using a focused energy source Limited material options	[18,22]
Powder Bed Fusion (PBF)	Implants Scaffolds (both in the discovery phase)	Use laser/electron beam to fuse powdered materials layer by layer with high accuracy and strength	[18,23,24,25]
Material Jetting (MJ)	Oral tablets (in the discovery phase, but expected to transition to clinical trials)	High resolution Deposits droplets of material solidified using UV light Good for details structures	[18,26,27]
Selective Laser Sintering (SLS)	Drug delivery devices Implants	Fuses powdered materials layer by layer using a laser suitable for robust and complex drug delivery systems	[18,23,28]
Selective Laser Melting (SLM)	Implants Scaffolds	Similar to SLS, but fully melts the powder resulting in denser structures	[18,24]
Inkjet Printing (IJ)	Oral tablets Implants	High precision Uses liquid ink to build layers suitable for intricate designs	[18,28,29]
3D-Printed Polypills	Multi-drug tablets	Combines multiple drugs into the pill Tailored release profiles Enhances patient compliance	[9,30]
Biodegradable Stents	Cardiovascular applications	Local drug delivery Controlled release Reduces systemic side effects Supports tissue regeneration	[19,20]
Microneedles	Pain-free drug delivery	Dissolvable after penetration Direct drug release into the bloodstream Minimal patient discomfort	[20]
3D-printed bone scaffolds	Bone repair Osteomyelitis treatment	Combines structural support with localized drug delivery customized to the patient’s anatomy	[18,19]
Customized drug delivery systems	Patient-specific implants	Personalized implants with integrated drug delivery tailored to individual patients	[22,26]

## Data Availability

Data sharing does not apply to this article as no new data were created or analyzed in this study.
